# Integrated Transcriptomic and Single-Cell Analysis Reveals Shared Macrophage-Associated Inflammatory Signatures in MASLD/NAFLD and Rheumatoid Arthritis

**DOI:** 10.3390/ijms27209150

**Published:** 2026-10-15

**Authors:** Wenbo Yu, Qianqian Nie, Yanze Wu, Xintian Lan, Jing Liu, Duoduo Xu, Shuguang Guan

**Affiliations:** College of Pharmacy, Changchun University of Chinese Medicine, Changchun 130117, China

**Keywords:** metabolic dysfunction-associated steatotic liver disease, nonalcoholic fatty liver disease, rheumatoid arthritis, macrophages, S100A8/A9, single-cell RNA sequencing, triptolide

## Abstract

Metabolic dysfunction-associated steatotic liver disease/nonalcoholic fatty liver disease (MASLD/NAFLD) and rheumatoid arthritis (RA) may share inflammatory mechanisms, although their common cellular basis remains unclear. We integrated bulk transcriptomic datasets (GSE66676 and GSE55235), single-cell RNA-sequencing datasets from murine RA synovium (GSE192504) and NASH liver (GSE129516), disease-association analysis, molecular docking, and in vitro experiments in LPS-stimulated RAW264.7 macrophages. Bulk transcriptomic analysis identified 630 shared differentially expressed genes, including 344 concordantly upregulated genes enriched in immune response, cytokine and chemokine signaling, phagosome, Toll-like receptor signaling, and Fc receptor-mediated phagocytosis; a strict dual-dataset intersection further defined 18 core genes significantly upregulated in both diseases. Immune-signature analysis indicated altered myeloid cell-associated inflammatory activity in both diseases, and several shared genes, including S100A8, S100A9, FCER1G, FCGR3A, LYZ, and MS4A7, correlated positively with monocyte/macrophage-related signatures. Single-cell analysis identified macrophage subsets with high S100a9 expression and elevated shared myeloid inflammatory signature scores in both RA synovium and NASH liver. Triptolide showed favorable predicted binding to the S100A8/A9 complex and reduced LPS-induced inflammatory cytokine secretion and expression of the six-gene signature in RAW264.7 cells, supporting the hypothesis that S100A9-associated inflammatory macrophage activation represents a candidate target for further investigation. These findings support a shared S100A9-positive inflammatory macrophage-associated program in MASLD/NAFLD and RA, and support the hypothesis that the S100A8/A9 complex represents a candidate target for further investigation.

## 1. Introduction

Metabolic dysfunction-associated steatotic liver disease (MASLD) is one of the most prevalent chronic liver diseases worldwide. Its spectrum ranges from simple steatosis to metabolic dysfunction-associated steatohepatitis (MASH), fibrosis, cirrhosis, and hepatocellular carcinoma [1,2]. MASLD is closely associated with obesity, insulin resistance, type 2 diabetes, and dyslipidemia, and it contributes to an increased risk of extrahepatic cardiometabolic complications [3]. Its development and progression are driven by interconnected mechanisms, including lipid dysregulation, lipotoxicity, oxidative stress, gut-derived signals, and immune-inflammatory responses [4]. Liver macrophages, comprising resident Kupffer cells and monocyte-derived macrophages, are central to hepatic homeostasis and inflammatory regulation. Persistent macrophage activation can promote inflammatory mediator release, immune-cell recruitment, and fibrogenesis, thereby contributing to disease progression [5,6].

Rheumatoid arthritis (RA) is a chronic systemic autoimmune disease characterized by persistent synovitis, progressive cartilage and bone destruction, and impaired joint function [7]. Its pathogenesis involves genetic susceptibility, environmental exposures, and dysregulated immune responses. Interactions among immune and stromal cells in the synovium establish a pro-inflammatory microenvironment characterized by sustained cytokine, chemokine, and immune receptor signaling [8]. Macrophages are important effector cells in RA synovitis, and their abundance and activation states are associated with disease activity. By producing tumor necrosis factor-alpha, interleukin-1 beta, interleukin-6, and other inflammatory mediators, activated macrophages contribute to inflammatory amplification, synovial hyperplasia, and structural joint damage [9,10].

Although MASLD and RA primarily affect different organs, both are associated with chronic low-grade inflammation, metabolic disturbances, and immune dysregulation. Patients with RA frequently present with obesity, insulin resistance, dyslipidemia, and abnormal liver function, while chronic systemic inflammation and treatment-related factors may further influence hepatic metabolism and injury [11,12]. Conversely, MASLD is increasingly recognized as a multisystem metabolic disorder accompanied by systemic inflammation and elevated extrahepatic disease risk [3,4]. Myeloid-cell activation, chemokine-mediated cell recruitment, pattern-recognition receptor signaling, and phagocytic processes may therefore represent shared immunoinflammatory features of the two diseases. However, their common transcriptomic profiles, relevant myeloid-cell subsets, and potential regulatory networks have not been comprehensively defined. Recent single-cell multi-omics investigations have reshaped our understanding of macrophage biology in metabolic and autoimmune disorders, uncovering extensive phenotypic heterogeneity and immunometabolic reprogramming that underpin chronic inflammatory progression [13,14,15,16,17]. Emerging work has described disease-specific macrophage functional states, such as lipid-associated macrophages in MASLD and metabolically rewired synovial macrophage subsets in RA. Nevertheless, cross-disease comparative studies that systematically map shared transcriptomic signatures and further assign these molecular profiles to discrete macrophage subpopulations across MASLD and RA remain scarce. This represents a critical knowledge gap: while individual-disease macrophage signatures are increasingly documented, it is still unclear whether MASLD and RA converge on conserved myeloid transcriptional programs confined to particular macrophage subsets.

Advances in bulk and single-cell RNA sequencing enable characterization of disease-associated transcriptional changes, immune microenvironments, and cellular heterogeneity at complementary levels. In this study, we integrated public bulk transcriptomic datasets from MASLD/NAFLD and RA to identify shared differentially expressed genes and performed functional enrichment, immune-infiltration, and disease-association analyses. We further used single-cell RNA-sequencing datasets from RA synovium and NASH liver to investigate the expression patterns of shared myeloid inflammatory signatures across macrophage subsets. Triptolide was selected as the candidate molecule for subsequent validation through a stepwise screening approach. Candidate compounds were initially identified from gene–disease–compound association databases based on their reported links to the shared inflammatory genes and the S100A8/A9 complex, and were further prioritized by molecular docking against the S100A8/A9 heterodimer. Among the candidates, triptolide exhibited the most favorable docking score and was therefore selected for functional validation in an LPS-induced macrophage inflammatory model. This study aimed to characterize shared inflammatory transcriptional programs between MASLD/NAFLD and RA and to provide evidence for future mechanistic and therapeutic investigations.

## 2. Results

### 2.1. Integrated Transcriptomic Analysis Identifies Differentially Expressed Genes in MASLD/NAFLD and RA

**Figure 1:**
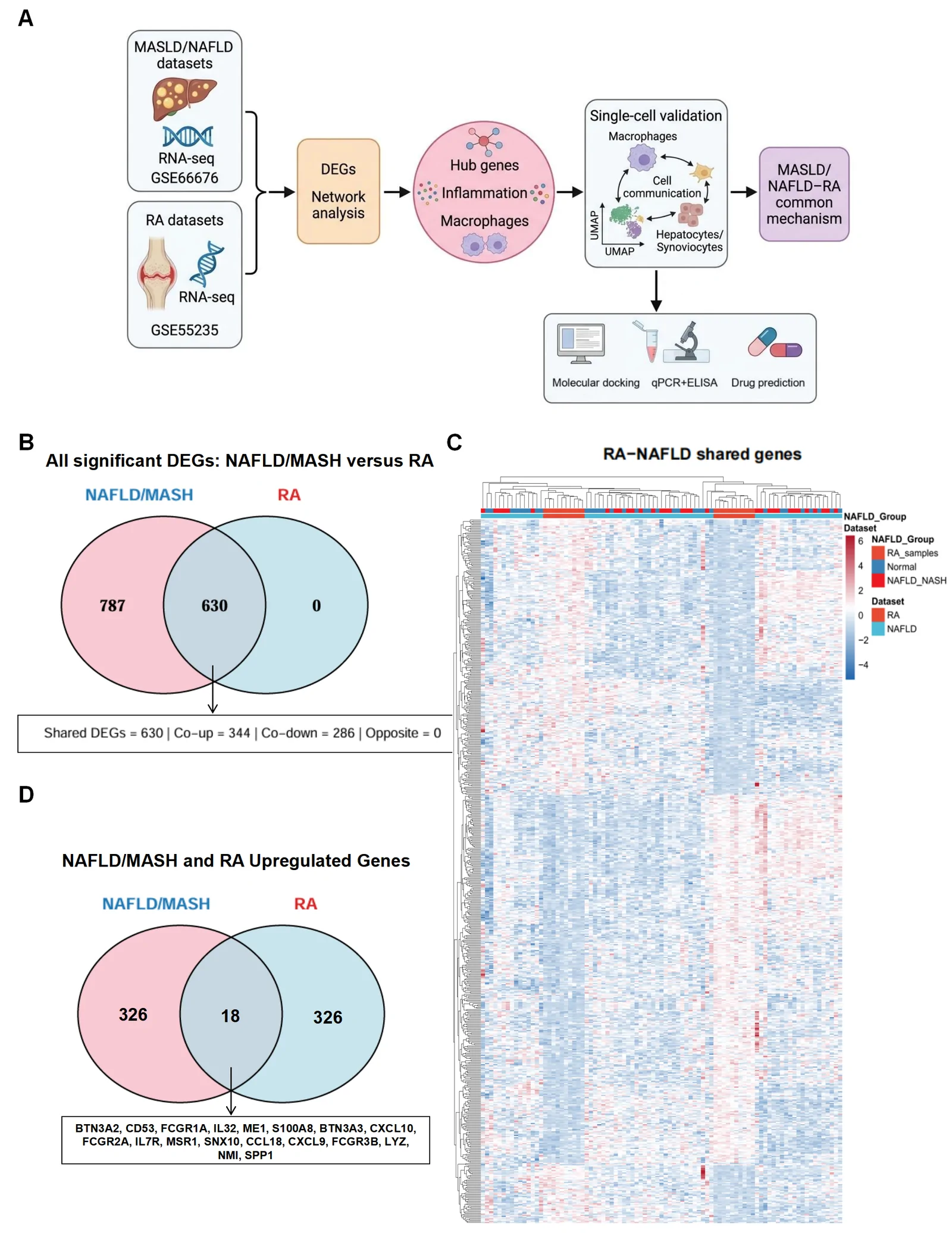
Integrated transcriptomic analysis identifies shared differentially expressed genes between MASLD/NAFLD and RA. (**A**) Schematic overview of the study design. Bulk RNA-seq/transcriptomic datasets from MASLD/NAFLD and RA were integrated to identify shared differentially expressed genes (DEGs), followed by network analysis, hub gene screening, single-cell validation, external validation, and drug prediction to explore a potential macrophage-mediated inflammatory mechanism linking MASLD/NAFLD and RA. Created with BioGDP.com (https://BioGDP.com). (**B**) Venn diagram of the 630 shared DEGs identified by intersecting the DEG lists of the two datasets, showing 344 concordantly upregulated and 286 concordantly downregulated genes. (**C**) Heatmap visualization showing expression patterns of 630 shared DEGs across individual disease and control samples from MASLD/NAFLD and RA datasets. (**D**) Venn diagram of the strict intersection of the upregulated DEG lists computed independently in each dataset (|log2FC| ≥ 1, adjusted *p*-value < 0.05 in both), yielding 18 core genes concurrently upregulated in both diseases, including S100A9, CCL3, FCER1G, LILRB2, MS4A7, S100A8, S100A12, CXCL10, FCGR2A, LTB, SPI1, SNX10, NMI, CCL18, CXCL9, FCGR3A, LYZ, and SPP1. These 18 genes are a subset of the 344 concordantly upregulated genes.

To investigate the potential shared molecular basis between metabolic dysfunction-associated steatotic liver disease/metabolic dysfunction-associated steatohepatitis (MASLD/NAFLD) and rheumatoid arthritis (RA), we performed an integrated analysis of the MASLD/NAFLD-related transcriptomic dataset GSE66676 and the RA-related transcriptomic dataset GSE55235. The overall analytical workflow is shown in Figure 1A. This workflow included differential expression analysis, cross-disease gene intersection, network analysis, single-cell validation, and external validation, with the aim of identifying shared transcriptomic features and potential macrophage-associated inflammatory mechanisms between the two diseases.

Differential expression analysis revealed substantial disease-associated transcriptional changes in both the MASLD/NAFLD and RA datasets. Cross-disease comparison identified 630 shared DEGs by intersecting the DEG lists computed independently within each dataset (|log2FC| ≥ 1, adjusted *p*-value < 0.05). Among them, 344 genes were concordantly upregulated and 286 genes concordantly downregulated; no gene showed opposite expression directions between the two diseases (Figure 1B). These findings suggest that the transcriptional overlap between MASLD/NAFLD and RA is characterized predominantly by concordant rather than opposing gene-expression changes.

To further assess the expression patterns of the shared DEGs across individual samples, we generated a heatmap based on the common gene set. The shared DEGs displayed distinct expression patterns between disease and control samples and showed a relatively consistent direction of change across the two datasets (Figure 1C). These results support the possibility that the overlapping genes represent common disease-associated molecular signatures shared by MASLD/NAFLD and RA.

We next focused on genes consistently upregulated in both MASLD/NAFLD and RA. To this end, we performed a strict intersection of the upregulated DEG lists computed independently within each dataset, retaining genes satisfying |log2FC| ≥ 1 and adjusted *p*-value < 0.05 in both datasets simultaneously. This yielded 18 core genes (Figure 1D), which constitute a subset of the 344 concordantly upregulated genes and were obtained without additional network-based or ranking-based filtering. Several of these genes, including S100A8, S100A9, S100A12, CCL3, CXCL9, CXCL10, FCGR2A, FCGR3A, and SPI1, are associated with myeloid-cell activation, inflammatory chemotaxis, cytokine signaling, and innate immune regulation. This expression pattern suggests that the shared upregulated genes may be closely related to monocyte/macrophage-associated inflammatory responses.

Taken together, the integrated transcriptomic analysis demonstrated that MASLD/NAFLD and RA share a substantial set of concordantly dysregulated genes. The enrichment of myeloid-cell- and inflammation-related genes among the shared upregulated genes provides an initial molecular basis for subsequent functional enrichment, immune infiltration, PPI network, machine-learning, and single-cell analyses. However, these findings primarily indicate a shared transcriptional association and do not, by themselves, establish a causal relationship or confirm a macrophage-mediated mechanism.

### 2.2. Functional Enrichment Analysis Reveals Immune-Inflammatory Pathways Shared by MASLD/NAFLD and RA

**Figure 2:**
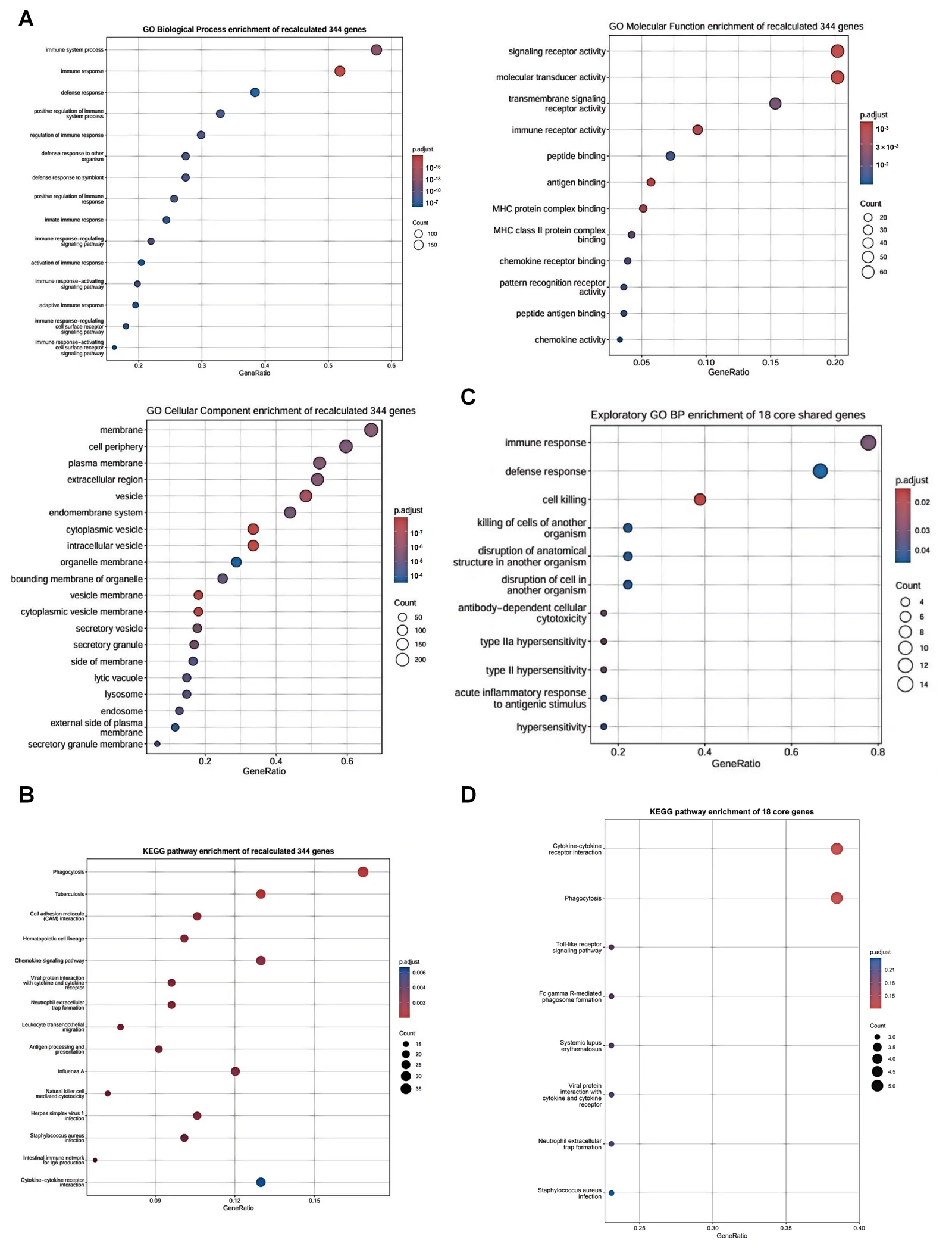
Functional enrichment analysis of shared upregulated genes in MASLD/NAFLD and RA. (**A**) Gene Ontology (GO) enrichment analysis of the 344 genes commonly upregulated in the MASLD/NAFLD and RA datasets. The enriched terms are presented separately for biological process, molecular function, and cellular component categories. The dot color represents the adjusted *p* value, and dot size indicates the number of genes associated with each GO term. (**B**) Kyoto Encyclopedia of Genes and Genomes (KEGG) pathway enrichment analysis of the 344 commonly upregulated genes. The dot color represents the adjusted *p* value, and dot size indicates the number of genes enriched in each pathway. (**C**) Exploratory GO enrichment analysis of the 18 genes commonly upregulated in both MASLD/NAFLD and RA. The dot color represents the adjusted *p* value, and dot size indicates the number of genes associated with each enriched GO term. (**D**) KEGG pathway enrichment analysis of the 18 shared upregulated genes. The dot color represents the adjusted *p* value, and dot size indicates the number of genes contributing to each enriched pathway.

To further characterize the potential biological functions of the 344 commonly upregulated genes, we performed Gene Ontology (GO) and Kyoto Encyclopedia of Genes and Genomes (KEGG) enrichment analyses. As shown in Figure 2A, the enriched biological processes were mainly related to immune responses, immune effector processes, leukocyte-mediated immune responses, and cytokine-associated signaling. The enriched molecular functions included receptor activity, signal receptor regulation, cytokine receptor binding, chemokine receptor binding, and immune receptor binding, whereas the cellular component terms were primarily associated with the cell membrane, cell surface, plasma membrane, vesicles, and extracellular vesicles. Collectively, these findings indicate that the shared upregulated genes are involved in cell-surface immune recognition, inflammatory mediator signaling, and immune cell communication, rather than being restricted to a single metabolic process.

KEGG analysis further defined the pathway characteristics of these shared genes (Figure 2B). The 344 commonly upregulated genes were significantly enriched in cytokine–cytokine receptor interaction, chemokine signaling, phagosome, Toll-like receptor signaling, and Fcγ receptor-mediated phagocytosis. These pathways represent coordinated processes involving inflammatory cell recruitment, innate immune recognition, immune-complex processing, and phagocytic activity. Therefore, the transcriptional overlap between MASLD/NAFLD and RA may reflect an interconnected pattern of inflammatory-cell recruitment and myeloid-cell activation. This interpretation is also consistent with the concurrent upregulation of S100A8, S100A9, S100A12, FCER1G, FCGR2A, FCGR3A, LYZ, and SPP1, suggesting that monocyte/macrophage-associated immune activity may contribute substantially to the shared inflammatory phenotype of the two diseases.

To obtain a more specific functional interpretation, we next performed exploratory GO and KEGG analyses of the 18 genes that were commonly upregulated in both MASLD/NAFLD and RA. These genes were mainly associated with immune response, defense response, cell killing, antibody-dependent cellular cytotoxicity, cytotoxicity, and acute inflammatory response (Figure 2C). KEGG analysis showed enrichment in cytokine–cytokine receptor interaction, phagosome, Toll-like receptor signaling, and Fc receptor-related pathways (Figure 2D). Compared with the broader enrichment profile of the 344-gene set, the 18-gene signature was more specifically concentrated in phagocytic activity, immune receptor signaling, and inflammatory effector functions, further supporting its association with activated monocyte/macrophage-related programs. Overall, the results shown in Figure 2 suggest that MASLD/NAFLD and RA may share a molecular network characterized by myeloid-cell activation, chemokine-mediated immune-cell recruitment, and immune-receptor-driven amplification of inflammation. However, these enrichment results represent statistical associations and cannot, by themselves, establish a macrophage-mediated mechanism or a causal relationship between the two diseases. Of note, enrichment results for the 18-gene core set should be considered exploratory. Owing to the limited number of input genes, statistical power is restricted, and these pathway associations require further independent validation.

### 2.3. Immune Signature Analysis Supports a Shared Myeloid-Cell-Associated Inflammatory State in MASLD/NAFLD and RA

**Figure 3:**
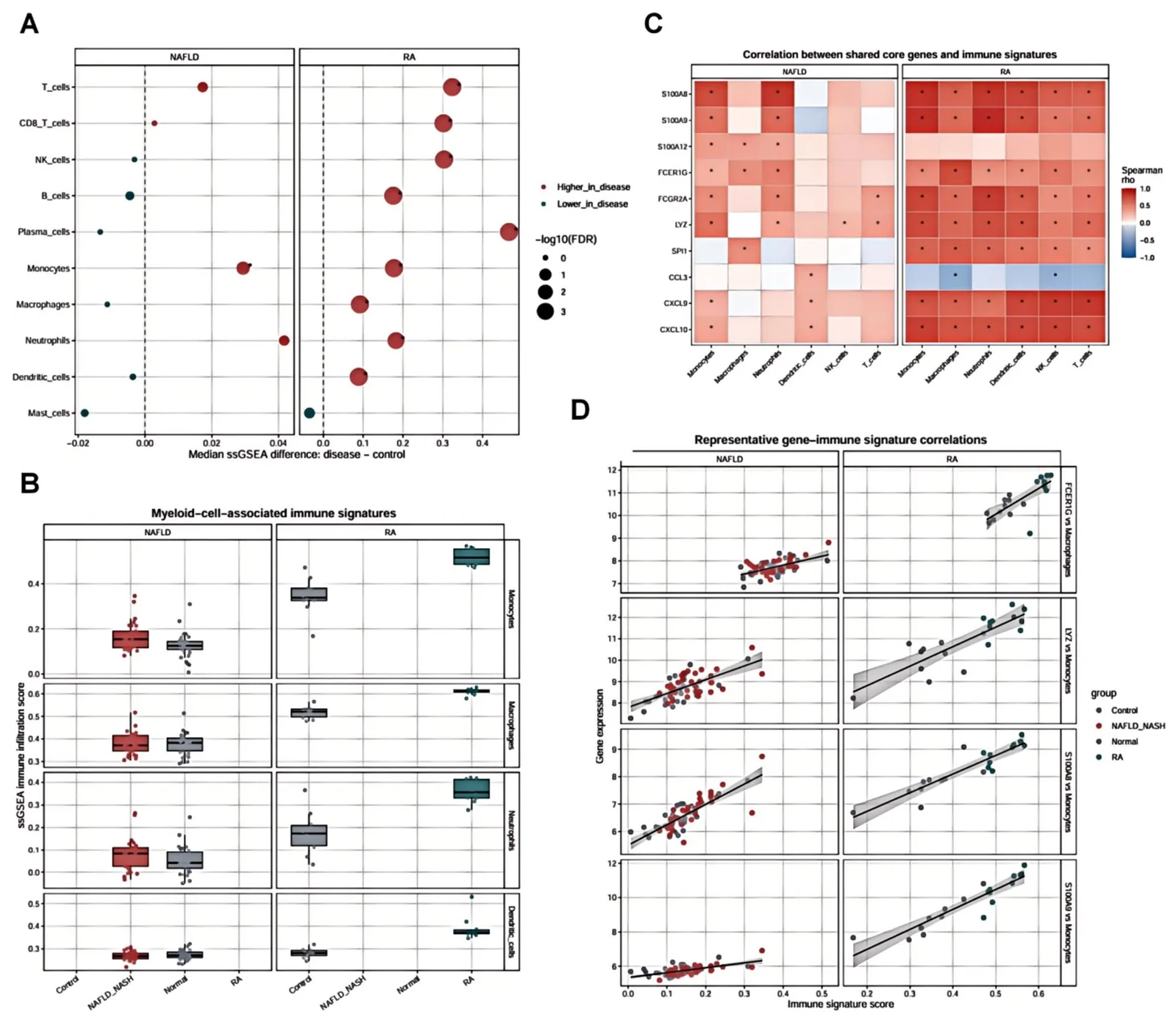
Immune signature analysis reveals shared myeloid-cell-associated inflammatory features in MASLD/NAFLD and RA. (**A**) Comparison of immune cell-related ssGSEA signatures between disease and control samples in the MASLD/NAFLD and RA datasets. The *x*-axis represents the median ssGSEA score difference between disease and control groups. Red points indicate signatures increased in disease samples, whereas blue-green points indicate signatures decreased in disease samples. Point size represents −log10(FDR). (**B**) Boxplots showing myeloid-cell-associated ssGSEA scores, including monocyte-, macrophage-, neutrophil-, and dendritic-cell-related signatures, in MASLD/NAFLD and RA datasets. Disease and control groups are shown separately, and statistical significance is indicated above each comparison. (**C**) Heatmap showing Spearman correlations between the shared core genes and immune cell-related ssGSEA signatures. Red indicates positive correlations, whereas blue indicates negative correlations. Asterisks denote statistically significant associations. (**D**) Representative scatter plots showing correlations between selected shared core genes and myeloid-cell-associated immune signatures in MASLD/NAFLD and RA. Each point represents an individual sample, and fitted regression lines with confidence intervals illustrate the relationship between gene expression and immune signature scores.

To further characterize the immune features associated with the shared DEGs, we performed ssGSEA-based analysis of immune cell-related transcriptional signatures in the MASLD/NAFLD and RA datasets. As shown in Figure 3A, both diseases exhibited alterations in immune signatures, although the specific patterns were not identical. The monocyte-related signature was most prominently increased in MASLD/NAFLD, whereas RA showed broader enhancement of T-cell, NK-cell, B-cell, plasma-cell, and several myeloid-cell-related signatures. These findings indicate that both diseases are associated with abnormalities in the immune microenvironment, but the immune alterations in RA appear to involve a broader range of immune cell-related transcriptional programs, while the shared features in MASLD/NAFLD may be more strongly concentrated in monocyte-related and other innate immune signals.

Given that the shared upregulated genes were mainly enriched in chemokine, phagosome, Toll-like receptor, and Fc receptor-related pathways, we next focused on myeloid-cell-associated signatures, including monocytes, macrophages, neutrophils, and dendritic cells (Figure 3B). We emphasize that ssGSEA scores from bulk tissue transcriptomes reflect transcriptional signature activity and cannot discriminate whether higher scores stem from greater immune-cell infiltration or from upregulated gene expression within resident tissue cells. The monocyte-related signature was markedly higher in MASLD/NAFLD disease samples than in controls, consistent with the concordant upregulation of myeloid-associated genes such as S100A8, S100A9, S100A12, and LYZ. RA showed a broader pattern of altered myeloid-cell-associated signatures, with increased scores observed in several monocyte-, macrophage-, neutrophil-, and dendritic-cell-related signatures. However, the magnitude and direction of changes among specific immune cell subsets were not completely consistent between the two diseases. Therefore, these findings are more appropriately interpreted as evidence of altered myeloid-cell-associated inflammatory activity and immune-cell recruitment rather than as evidence of a uniform increase in a particular cell population in both diseases.

We further examined the correlations between the 18 commonly upregulated core genes and immune cell-related signatures using Spearman correlation analysis (Figure 3C). Multiple core genes, including S100A8, S100A9, S100A12, FCER1G, FCGR3A, LYZ, and MS4A7, showed positive correlations with monocyte- and macrophage-related signatures. Representative gene–myeloid signature scatter plots showed similar positive trends in both the MASLD/NAFLD and RA datasets, with several associations reproducible across the two diseases (Figure 3D). These results support a close relationship between the shared upregulated transcriptional program and myeloid-cell-associated inflammatory activity, and suggest that enhanced myeloid-cell recruitment or activation, inflammatory chemokine production, and phagocytic and immune-receptor signaling may represent a common immunological link between MASLD/NAFLD and RA. Nevertheless, because this analysis was based on bulk transcriptomic data and gene-set scores, it cannot distinguish between increased immune-cell abundance and enhanced intracellular transcriptional activity. Therefore, the findings do not establish that macrophages mediate the shared disease mechanism, and the specific cellular origins and functional roles of these core genes require further validation using single-cell transcriptomic data.

### 2.4. Single-Cell Transcriptomics Identifies a Shared S100A9-Positive Inflammatory Macrophage Subset in MASLD/NAFLD and RA

**Figure 4:**
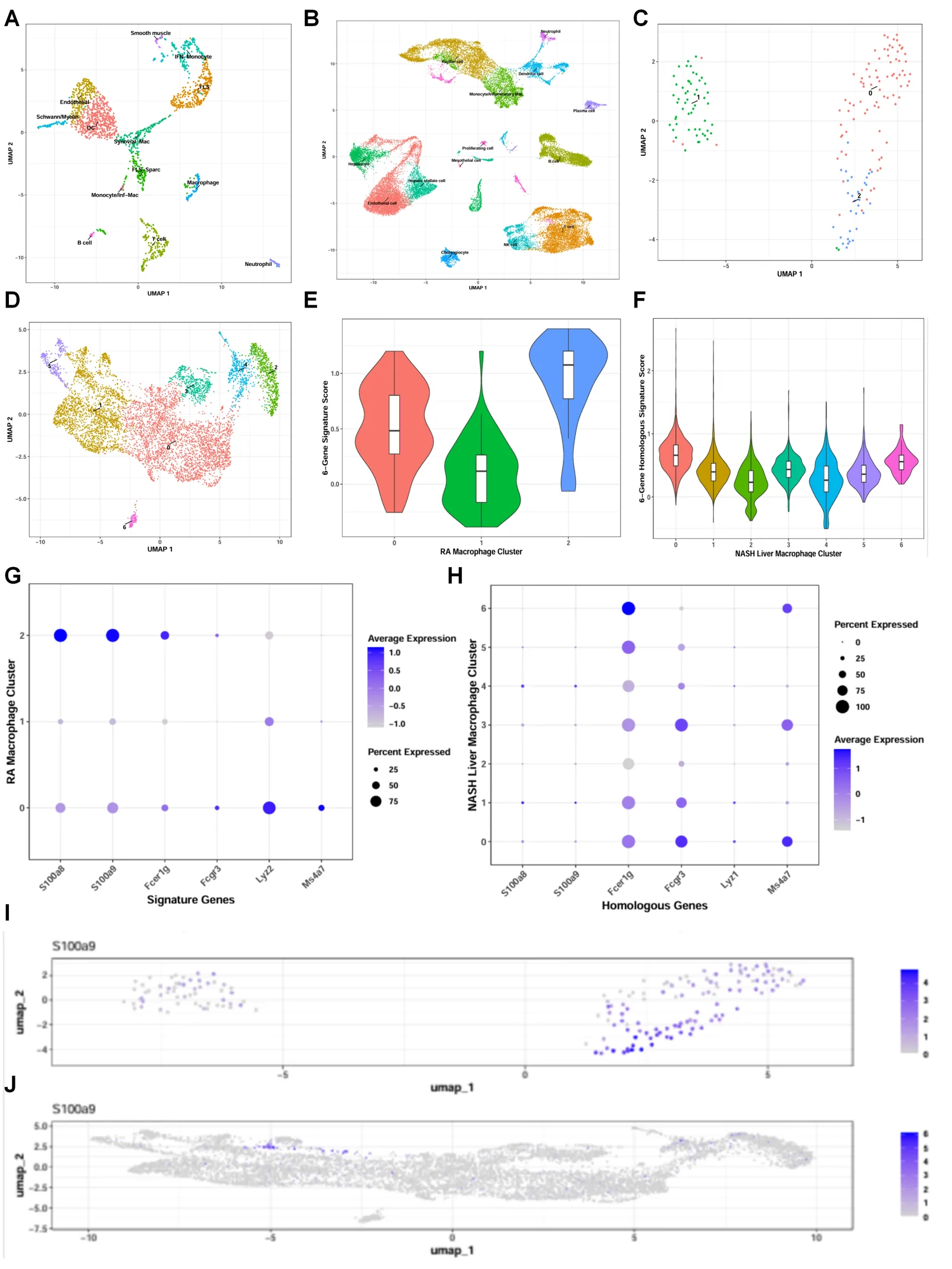
Single-cell transcriptomic profiling identifies a shared S100A9-positive inflammatory macrophage subset linking MASLD/NAFLD and RA. (**A**,**B**) Annotated UMAP visualizations of all cell populations in murine CIA-RA synovium (GSE192504, (**A**)) and murine NASH liver (GSE129516, (**B**)). Each color denotes a distinct cell type, annotated based on canonical marker genes. (**C**,**D**) UMAP plots showing macrophage subsets extracted from RA synovium (**C**) and NASH liver (**D**), followed by unsupervised re-clustering. Synovial macrophages were partitioned into three subclusters (**C**), whereas liver macrophages were partitioned into multiple subclusters (**D**). (**E**,**F**) Violin plots displaying module scores of the six-gene homologous inflammatory-macrophage signature across macrophage subclusters in RA synovium (**E**) and NASH liver (**F**). Higher scores indicate a stronger shared inflammatory-macrophage phenotype. (**G**,**H**) Dot plots showing the expression patterns of six signature genes across macrophage subclusters in RA (**G**) and NASH liver (**H**). Dot color represents average expression level; dot size indicates the proportion of cells expressing the given gene. (**I**,**J**) FeaturePlots illustrating the spatial distribution of S100a9 expression within RA synovial macrophages ((**I**), GSE192504) and NASH liver macrophages ((**J**), GSE129516). High S100a9 expression overlaps with macrophage clusters exhibiting elevated signature module scores in both datasets.

To trace the cellular origin of the shared myeloid inflammatory signature uncovered by bulk transcriptome analysis, we analyzed two publicly available single-cell RNA-seq datasets: murine CIA-induced rheumatoid arthritis synovium (GSE192504) and murine NASH liver (GSE129516). After quality control, dimensionality reduction, clustering and cell annotation, multiple major cell populations were identified in both datasets, including monocytes/macrophages, T cells, B cells, neutrophils and stromal cells (Figure 4A,B). Macrophage subsets were extracted separately from the two datasets and subjected to re-clustering. The synovial macrophages from RA mice were further partitioned into three subclusters (Figure 4C), while liver macrophages from NASH mice were divided into multiple subclusters (Figure 4D).

S100A12 was excluded from signature construction because it was undetectable in this RA single-cell dataset and lacks a mouse ortholog. We constructed a six-gene homologous signature (S100a8, S100a9, Fcer1g, Fcgr3, Lyz1/Lyz2, Ms4a7) and calculated module scores for each macrophage subcluster using the AddModuleScore function in Seurat. Violin plots revealed one macrophage cluster with markedly elevated signature scores in RA synovial macrophages (Figure 4E). Similarly, a macrophage subset with high signature scores was identified in the liver of NASH mice (Figure 4F).

Dot plot visualization demonstrated that among the six signature genes, S100a8 and S100a9 exhibited the highest average expression and the largest fraction of positive cells within these high-score macrophage clusters, while expression of Fcer1g, Fcgr3, Lyz1/Lyz2 and Ms4a7 was relatively moderate (Figure 4G,H). As a core component of the MRP8/MRP14 heterodimer and a well-recognized marker of activated inflammatory myeloid cells, S100a9 was selected as the representative gene for UMAP spatial visualization. Feature plots verified that cells with high S100a9 expression were predominantly enriched in macrophage clusters with elevated six-gene signature scores in both the RA synovium (GSE192504) and NASH liver (GSE129516) datasets (Figure 4I,J). To avoid circular reasoning caused by defining cell clusters using the same predefined signature used for module scoring, we further performed unbiased differential expression analysis comparing each high-signature-score macrophage cluster to other macrophage subsets within each dataset. De novo computed top marker genes included S100a8, S100a9, along with additional myeloid inflammatory genes. Functional enrichment of these unbiased marker genes highlighted pathways related to innate immune activation, cytokine response and phagocytosis, further confirming the inflammatory nature of these S100A9-positive macrophage populations.

Collectively, single-cell transcriptomic analysis demonstrates that MASLD/NAFLD and RA share a population of S100A9-positive inflammatory macrophages carrying this conserved myeloid signature. These findings suggest that S100A9-dependent macrophage activation may serve as a common cellular basis underlying shared pathogenesis of the two diseases.

### 2.5. Disease Association Characteristics of Shared Myeloid Inflammatory Genes and Molecular Docking Validation of Candidate Small Molecules Targeting the S100A8/A9 Complex

**Figure 5:**
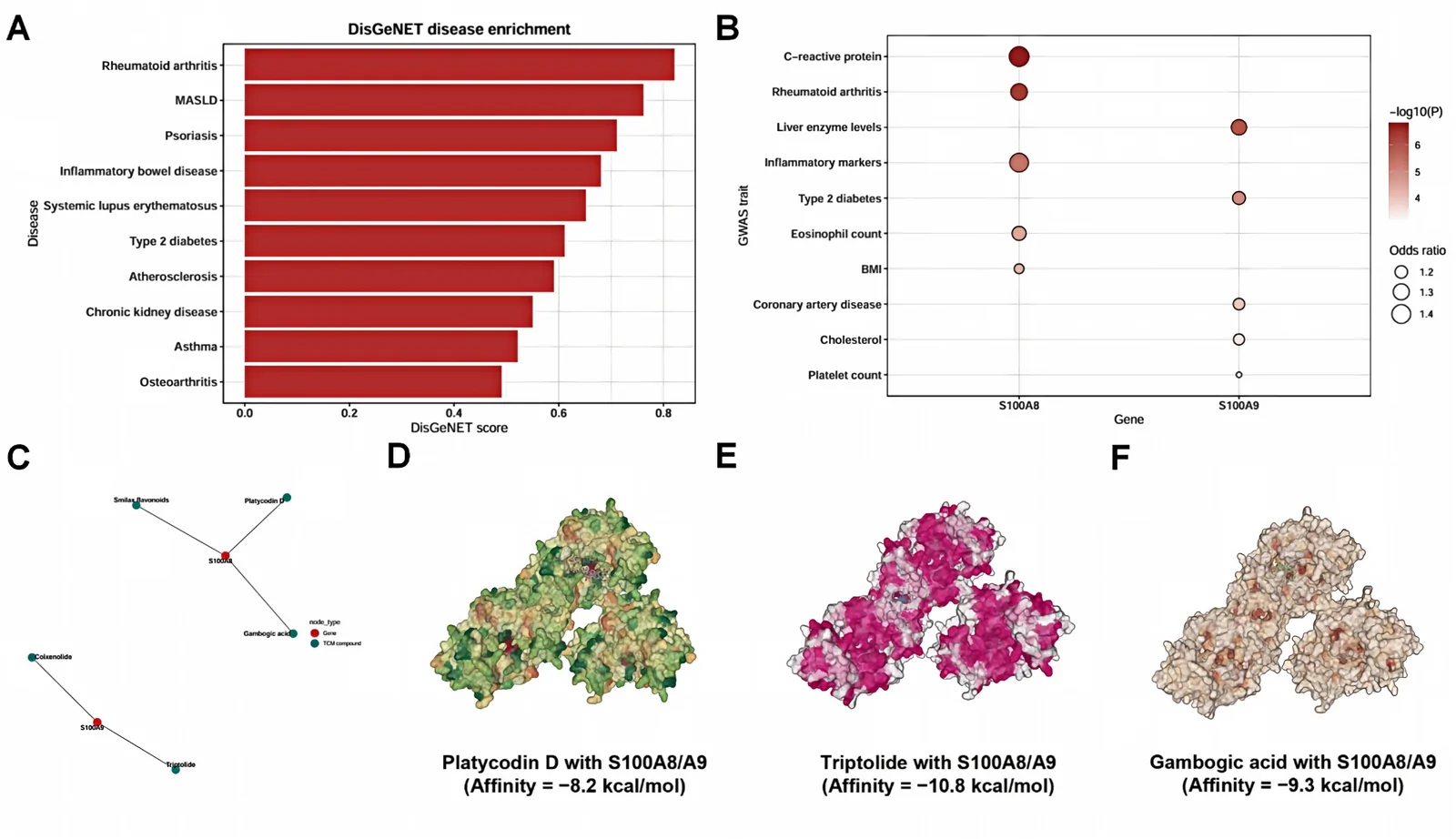
Disease association analysis of shared inflammatory genes and molecular docking of small molecules targeting the S100A8/A9 complex. (**A**) Bar plot of DisGeNET disease enrichment, showing association scores and enrichment significance of shared inflammatory genes across various diseases. (**B**) GWAS phenotype association scatter plot illustrating the association effect and significance of S100A8 and S100A9 loci with diverse clinical phenotypes (color gradient represents −log10(P), and dot size indicates the odds ratio). (**C**) Gene–disease–compound association network. Core nodes show the connections between S100A8/A9, disease phenotypes and candidate small-molecule compounds. (**D**–**F**) 3D molecular docking binding modes of platycodin D (**D**), triptolide (**E**), and gambogic acid (**F**) within the binding pocket of the S100A8/A9 heterodimer. Corresponding docking-score values from molecular-docking simulation are annotated for each compound.

To systematically dissect the clinical disease associations of this shared myeloid inflammatory signature and explore potential small-molecule candidates targeting the core node S100A8/A9 heterodimer, we performed enrichment analysis and molecular docking simulation by integrating multi-omics association databases. DisGeNET-based disease enrichment analysis revealed that the shared inflammatory genes identified in this study exhibited the highest enrichment scores in rheumatoid arthritis and MASLD (formerly termed NAFLD), which was highly consistent with the shared macrophage phenotype observed in single-cell analysis (Figure 5A). GWAS phenotype association analysis further confirmed that genetic loci of S100A8 and S100A9 were significantly associated with serum C-reactive protein levels, genetic susceptibility to rheumatoid arthritis, abnormal liver function and other inflammation- and metabolism-related clinical phenotypes (Figure 5B). Of note, these DisGeNET and GWAS-derived associations provide contextual genetic background and cannot be regarded as independent experimental validation of our gene signature. Visualization of the gene–disease–compound association network demonstrated that S100A8/A9 acted as a core hub node connecting multiple inflammatory disease phenotypes and candidate natural small molecules, indicating its potential as a common therapeutic target for both RA and MASLD (Figure 5C).

Subsequently, molecular docking simulations were carried out for three candidate small molecules using the crystal structure of the S100A8/A9 heterodimer (PDB:1XK4) as the receptor. All three compounds exhibited favorable docking poses within the calcium-binding pocket of the S100A8/A9 heterodimer. Triptolide achieved the most favorable docking score (−10.8 kcal/mol) among the three tested compounds according to rigid-body molecular-docking simulation, followed by gambogic acid (−9.3 kcal/mol) and platycodin D (−8.2 kcal/mol) (Figure 5D–F). Collectively, these results suggest that the S100A8/A9 complex represents a candidate functional node associated with the shared inflammatory response in RA and MASLD, and natural small molecules such as triptolide warrant further investigation as potential modulators of the S100A9-positive inflammatory macrophage program. Direct target engagement of S100A8/A9 and the mechanistic dependence of triptolide’s effects on this complex remain to be experimentally established.

### 2.6. Triptolide Exerts Anti-Inflammatory Effects on LPS-Stimulated Inflammatory Macrophages In Vitro

To investigate the regulatory effect of triptolide on the activation of inflammatory macrophages, an in vitro inflammatory model was established by stimulating RAW264.7 murine macrophages with LPS, followed by intervention with gradient concentrations of triptolide. According to previous literature, the low, medium and high doses of triptolide were set as 5 nM, 10 nM and 20 nM, respectively.

**Figure 6:**
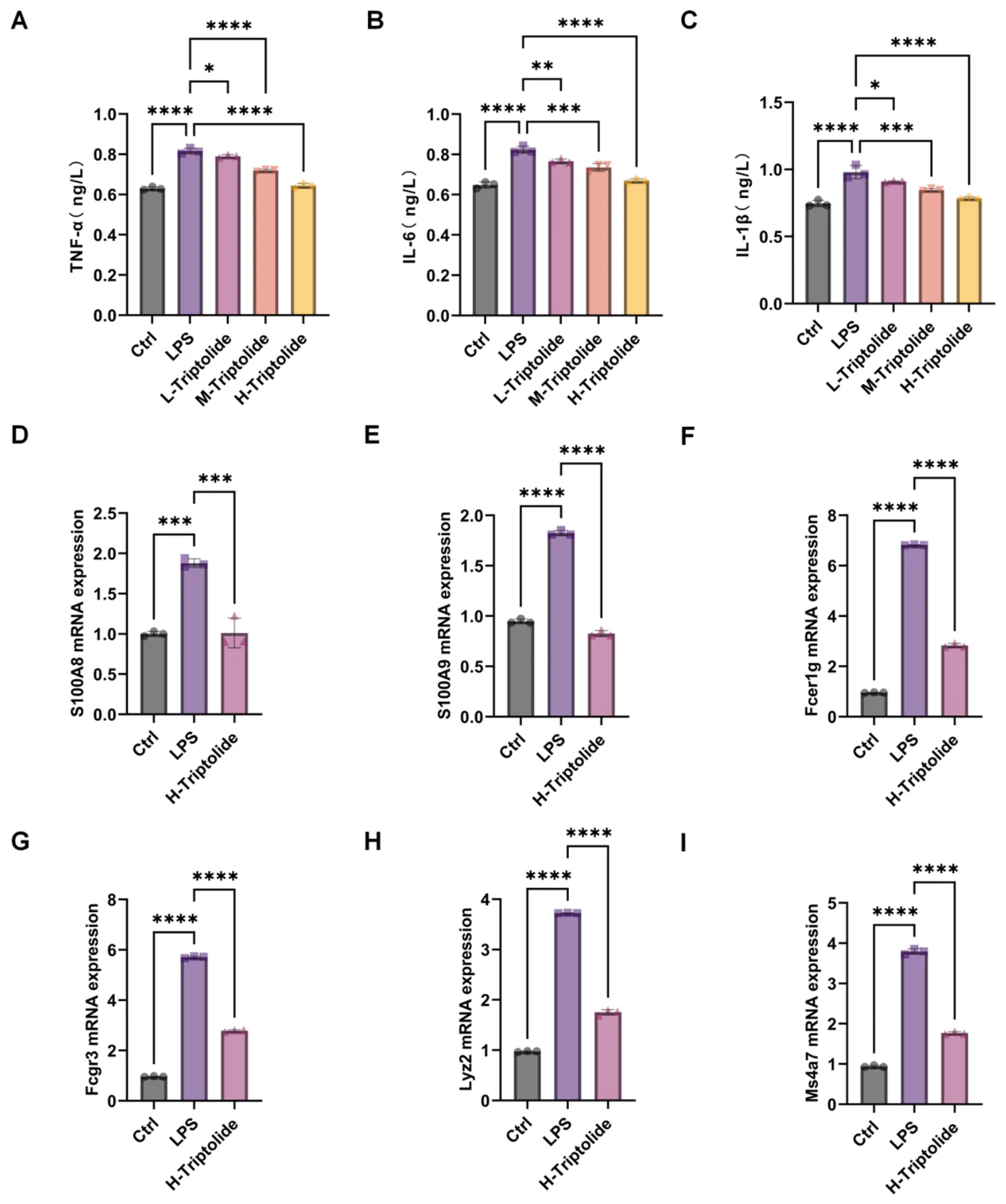
The in vitro anti-inflammatory effects of triptolide in inflammatory macrophages. (**A**–**C**) ELISA measurement of protein levels of TNF-α, IL-6 and IL-1β in cell culture supernatant from control (Ctrl), LPS model group, and groups treated with low-dose (5 nM, L-Triptolide), medium-dose (10 nM, M-Triptolide) and high-dose (20 nM, H-Triptolide) triptolide. (**D**–**I**) qRT-PCR analysis of mRNA relative expression of six shared inflammatory signature genes (S100a8, S100a9, Fcer1g, Fcgr3, Lyz2, Ms4a7) in macrophages from each group. Data are presented as mean ± SEM; * *p* < 0.05, ** *p* < 0.01, *** *p* < 0.001, **** *p* < 0.0001.

ELISA results demonstrated that LPS stimulation markedly induced the secretion of pro-inflammatory cytokines TNF-α, IL-6 and IL-1β in macrophages. After treatment with 5 nM, 10 nM and 20 nM triptolide, the secretion levels of pro-inflammatory cytokines decreased in a dose-dependent manner, and the maximal inhibitory effect was observed at the high dose of 20 nM triptolide (Figure 6A–C).

qRT-PCR was further performed to detect the transcriptional expression of the six shared myeloid inflammatory signature genes. The mRNA levels of S100a8, S100a9, Fcer1g, Fcgr3, Lyz2 and Ms4a7 were significantly upregulated upon LPS stimulation. Treatment with 20 nM triptolide robustly reduced the mRNA expression of all six signature genes (Figure 6D–I).

Collectively, these in vitro data indicate that triptolide suppresses LPS-induced pro-inflammatory cytokine release in a dose-dependent manner and downregulates the six-gene inflammatory signature shared between RA and MASLD. These effects are consistent with, but do not by themselves demonstrate, a direct action of triptolide on S100A9-positive inflammatory macrophages or on the S100A8/A9 complex.

## 3. Discussion

In this study, integrated bulk transcriptomic analyses of MASLD/NAFLD and RA identified shared differentially expressed genes that were predominantly enriched in immune-inflammatory processes, including cytokine and chemokine signaling, Toll-like receptor signaling, phagosome-related pathways, and Fc receptor-mediated phagocytosis. Immune-infiltration and single-cell RNA-sequencing analyses further suggested that monocyte/macrophage-associated inflammatory programs represent an important shared immunopathological feature of MASLD/NAFLD and RA. The progression of MASLD involves not only hepatocellular lipid accumulation but also lipotoxicity, oxidative stress, gut-derived inflammatory signals, and immune-cell infiltration [4,18]. Kupffer cells and monocyte-derived macrophages can recognize damage-associated and microbial-associated signals and subsequently promote hepatic inflammation, immune-cell recruitment, and fibrosis through the production of inflammatory mediators such as TNF-alpha, IL-1beta, IL-6, and CCL2 [5,6]. Single-cell studies have also identified macrophage subsets in NASH livers with inflammatory, lipid-processing, and pro-fibrotic characteristics [19].

RA synovium likewise exhibits prominent myeloid-cell activation and macrophage heterogeneity. Activated synovial macrophages produce inflammatory cytokines and chemokines and interact with fibroblast-like synoviocytes, T cells, B cells, and neutrophils to establish a persistent inflammatory network that promotes synovial hyperplasia, cartilage destruction, and bone erosion [7,8]. Previous studies have shown that synovial macrophage abundance is associated with RA disease activity and tissue damage and is reduced following effective treatment [20]. In the present study, shared myeloid inflammatory signatures were mainly distributed among macrophage-related subsets in both RA synovium and NASH liver single-cell datasets, suggesting that macrophages may provide a common cellular basis linking hepatic metabolic inflammation and autoimmune joint inflammation. Importantly, bulk transcriptomic data reflect overall tissue-level expression changes and cannot fully distinguish altered cellular composition from altered gene expression within individual cell types. The incorporation of single-cell datasets therefore improved interpretation of the shared molecular signatures and their potential cellular origins.

The shared genes identified in this study, including S100A8, S100A9, FCER1G, FCGR3A, LYZ, and MS4A7, are mainly associated with monocyte/macrophage activation, migration, phagocytosis, and inflammatory signaling. S100A8 and S100A9 encode calcium-binding proteins that form a heterodimer and function as damage-associated molecular patterns. Through receptors such as TLR4 and the receptor for advanced glycation end products, S100A8/A9 can activate downstream inflammatory pathways, including NF-kappaB signaling, thereby promoting cytokine production and immune-cell recruitment [21,22]. Elevated S100A8/A9 expression has been reported in RA synovium, peripheral blood, and synovial fluid and is associated with inflammation and disease activity [23]. In fatty liver disease, S100A8/A9-related myeloid cells may also contribute to the inflammatory and fibrotic hepatic microenvironment. In addition, FCER1G and FCGR3A participate in myeloid immune-receptor and Fc receptor signaling, whereas LYZ and MS4A7 are commonly associated with monocyte/macrophage phenotypes [24]. These findings support the presence of a shared transcriptional abnormality centered on myeloid immune activation in MASLD/NAFLD and RA; however, they do not establish that these genes directly drive the development or progression of either disease.

Molecular docking suggested a potential interaction between triptolide and the S100A8/A9 complex. In an LPS-induced RAW264.7 macrophage inflammatory model, triptolide reduced inflammatory cytokine levels and downregulated the expression of several shared myeloid inflammation-related genes. Previous studies have demonstrated that triptolide exerts anti-inflammatory and immunomodulatory effects through pathways such as NF-kappaB and MAPK signaling [25]. Nevertheless, molecular docking provides only computational evidence of a possible binding mode and cannot demonstrate direct target engagement in cellular or in vivo settings, nor can it establish that the anti-inflammatory effects of triptolide depend on S100A8/A9. Moreover, RAW264.7 cells and LPS stimulation do not fully recapitulate the complex microenvironments of MASLD liver tissue or RA synovium. Triptolide also has a narrow therapeutic window and is associated with potential hepatotoxicity, nephrotoxicity, reproductive toxicity, and immunosuppressive effects [26]. Another important caveat comes from cross-species differences. Our single-cell analyses are based on murine CIA-RA and experimental NASH mouse models. Although these experimental models recapitulate major inflammatory phenotypes of human rheumatoid arthritis and MASLD, they cannot fully recapitulate the full cellular heterogeneity, disease progression spectrum, and clinical complexity seen in human patients. The absence of a direct mouse ortholog for human S100A12, which prevented its inclusion in our murine signature, already illustrates such cross-species discrepancies. Accordingly, conclusions drawn from mouse single-cell data should be regarded as testable mechanistic hypotheses, and independent validation in human clinical samples is necessary to confirm whether these myeloid inflammatory signatures are conserved in human RA and MASLD.

This study has several limitations that should be acknowledged. First, all transcriptomic analyses rely on publicly deposited datasets, which carry inherent dataset heterogeneity and potential batch effects even after integration and normalization. Second, there are inevitable interspecies differences between human and murine immune and hepatic biology; our in vitro functional validation was performed solely using the RAW264.7 murine macrophage cell line, and these findings cannot be directly extrapolated to human patients. Third, we lack dedicated clinical samples from patients diagnosed with both MASLD and RA, which restricts translational interpretation. Fourth, although we identified a shared myeloid inflammatory gene signature between MASLD and RA, this shared signature does not imply identical upstream or downstream regulatory mechanisms in the two diseases. Finally, our functional validation was limited to in vitro cell experiments, and further in vivo protein-level validation is required to confirm the biological effects of triptolide. Future studies should validate the functions of key genes in human clinical samples and MASLD and RA animal models, while direct drug-target interactions should be assessed using approaches such as surface plasmon resonance, cellular thermal shift assays, and gene knockdown or knockout experiments.

## 4. Materials and Methods

### 4.1. Acquisition and Preprocessing of Bulk Transcriptomic Datasets

Publicly available transcriptomic datasets related to MASLD/NAFLD and RA were obtained from the Gene Expression Omnibus (GEO) database. The MASLD/NAFLD dataset GSE66676 and the RA dataset GSE55235 were included in the bulk transcriptomic analysis. The corresponding clinical annotations and sample information were obtained from the GEO database. Gene expression matrices were normalized according to the original platform annotations. When necessary, probe identifiers were converted into official gene symbols, and probes mapping to the same gene were summarized using the probe with the highest expression level. Genes with low or undetectable expression across most samples were excluded before downstream analysis.

### 4.2. Differential Expression Analysis and Identification of Shared Genes

Differential expression analysis was performed separately for each dataset using the limma package of Bioconductor in R (limma version 3.54.2; R version 4.2.1, accessed on 10 September 2026). Expression matrices were normalized according to the original platform annotations. Log2 fold-changes and adjusted *p*-values were derived using limma’s empirical Bayes moderated t-statistics, with multiple-testing correction implemented via the Benjamini–Hochberg method. DEGs were screened using thresholds of |log2FC| ≥ 1 and adjusted *p*-value < 0.05.

All DEGs from the two datasets were intersected to obtain 630 shared DEGs. Of these shared DEGs, 344 genes showed an upregulated expression direction within the shared DEG set. To obtain genes with consistent upregulation in both datasets simultaneously, we further performed intersection on upregulated DEG lists of MASLD/NAFLD and RA. This strict two-dataset intersection identified 18 genes that were significantly upregulated in both MASLD/NAFLD and RA. Correspondingly, 286 genes were concordantly downregulated shared DEGs. Genes showing opposite expression directions between the two diseases were analyzed separately and were not included in the concordant shared-gene set.

Venn diagrams were generated to visualize gene overlaps, and heatmaps were plotted to show expression patterns of shared DEGs across individual samples. The 18 intersected genes were regarded as core candidate inflammatory genes, which were predominantly enriched in myeloid-cell activation, chemokine signaling, immune-receptor signaling and phagocytosis-related functional pathways. The 18-gene core set is a subset of the 344 concordantly upregulated genes and was derived by strict intersection of the upregulated DEG lists of the two datasets, without additional network-based or ranking-based filtering.

### 4.3. Functional Enrichment Analysis

Gene Ontology (GO) and Kyoto Encyclopedia of Genes and Genomes (KEGG) enrichment analyses [27] were performed for the 344 concordantly upregulated genes and the 18 strict intersecting core-gene set. GO annotation database version: org.Hs.eg.db v3.18.0; KEGG database version: release 112.0.

The whole human protein-coding gene universe derived from the corresponding annotation package was used as the background gene set for over-representation analysis.

*p*-values were adjusted for multiple testing using the Benjamini–Hochberg false discovery rate (FDR) method. Enriched terms with adjusted *p*-value < 0.05 were regarded as statistically significant. The number of input genes overlapping each enriched term was reported in visualization outputs.

Notably, enrichment analysis for the 18-gene set was regarded as exploratory only. Given the small input gene count, statistical power is limited, and these results should be interpreted with caution rather than treated as robust pathway evidence. Enrichment results were visualized using dot plots, where dot size represents the number of input genes annotated to each term, and dot color corresponds to −log10(adjusted *p*-value).

### 4.4. Immune-Signature and Correlation Analyses

Single-sample gene set enrichment analysis (ssGSEA) was applied to calculate immune-cell-related signature scores. Immune cell gene sets were retrieved from the Molecular Signatures Database (MSigDB, version v7.5.1, accessed on 10 September 2026), including curated gene signatures for monocytes, macrophages, neutrophils, dendritic cells, T cells, B cells, plasma cells, and natural killer cells.

Signature scores were computed for each sample to reflect the relative transcriptional activity of corresponding immune-cell-related programs. Differences in ssGSEA signature scores between disease and control groups were compared within each dataset.

Notably, ssGSEA scores derived from bulk transcriptomic data cannot biologically distinguish whether elevated signature values originate from increased immune-cell abundance within tissue or enhanced intracellular transcriptional activity of resident cells.

To investigate the relationship between the shared core genes and immune-cell-associated transcriptional activity, Spearman correlation analysis was performed between individual gene-expression levels and immune-signature scores. Correlation coefficients and corresponding significance values were calculated separately for the MASLD/NAFLD and RA datasets. Correlation heatmaps and scatter plots were generated to display the relationships between the shared genes and myeloid-cell-associated signatures.

### 4.5. Single-Cell RNA-Sequencing Analysis

Single-cell RNA-seq analysis was performed on two public datasets, including GSE192504 (CIA-induced RA mouse synovium, *n* = 4 mice) and GSE129516 (NASH mouse liver, n = 5 mice). Raw cell count matrices were imported into Seurat (v4.3.0). Cells with nFeature_RNA < 200 or >6000, or mitochondrial gene proportion > 10% were filtered out for quality control. After QC, 24,678 cells remained for downstream analysis. Gene expression values were normalized using the NormalizeData function with default log-normalization. Highly variable genes were identified, followed by scaling and principal component analysis (PCA). The top 30 PCs were selected for dimensional reduction. Graph-based clustering was conducted using FindNeighbors and FindClusters with a resolution of 0.5. Cell-type annotation was assigned based on canonical marker genes: Adgre1, Cd68 for macrophages; Cd3d, Cd3e for T cells; Cd19, Ms4a1 for B cells; S100a8, S100a9 for neutrophils; Col1a1, Pdgfra for stromal cells. Batch integration across samples within each dataset was implemented using IntegrateData to mitigate batch effects. Macrophage subsets were then extracted and re-clustered separately for subsequent analysis. After identifying macrophage subclusters with high six-gene module scores, unbiased differential expression analysis was performed using the FindMarkers function in Seurat. Each high-score inflammatory macrophage cluster was compared against all remaining macrophage subclusters within the same dataset to detect de novo cluster marker genes, without relying on the predefined six-gene signature. Genes with |avg_log2FC| > 0.25 and adjusted *p*-value < 0.05 were considered significant markers. Top marker genes were subjected to GO/KEGG enrichment analysis to characterize functional properties of these macrophage clusters.

### 4.6. Disease-Association Analysis and Candidate Compound Screening

Disease-gene enrichment analysis was performed using DisGeNET database. Gene–disease associations were retained with a DisGeNET GDA-score ≥ 0.2; both curated and text-mined evidence entries were included for enrichment calculation. For GWAS phenotype association analysis, summary associations were retrieved from the GWAS Catalog. Only genome-wide significant records (*P* < 5 × 10^−8^) were retained. Most underlying GWAS studies were predominantly based on European-ancestry cohorts; reported effect measures included odds ratio (OR) and beta-coefficient for quantitative traits.

It should be emphasized that DisGeNET and GWAS-Catalog associations only provide supportive functional-genetic context for our transcriptomic outputs and do not constitute independent experimental validation of our shared myeloid inflammatory gene signature. Candidate small molecules associated with the shared inflammatory genes and the S100A8/A9 complex were screened using gene–disease–compound association databases and literature-supported resources. The resulting gene–disease–compound relationships were visualized as an interaction network. Triptolide, gambogic acid, and platycodin D were selected for subsequent molecular docking analysis.

### 4.7. Molecular Docking Analysis

Molecular docking was performed using AutoDock 4.2.6 with the Lamarckian genetic algorithm (LGA). The receptor protein (S100A8/A9 heterodimer, PDB ID: 1XK4) and three small-molecule ligands (triptolide, gambogic acid, platycodin D) were prepared using AutoDockTools to assign Gasteiger partial charges and define protonation states at pH 7.4. Identical docking parameters were applied for all three compounds. The docking search box was centered at X = 12.8, Y = 21.4, Z = 35.6, with dimensions 60 × 60 × 60 Å and a grid spacing of 0.375 Å. This calcium-binding pocket region was selected as the putative ligand-binding pocket, because it represents the reported functional interaction interface of S100A8/A9 that mediates binding to partner molecules and downstream inflammatory signaling. A total of 100 independent docking runs were executed for each compound, and 10 ligand poses were generated per run. The optimal pose for each compound was selected according to the lowest estimated docking energy together with geometric compatibility within the binding pocket.

Note that the docking score here represents empirical scoring-function output from rigid-body docking. It does not correspond to absolute thermodynamic binding free energy. This simulation only predicts static protein–ligand binding conformations and does not include molecular-dynamics-based free-energy calculation. Docking poses were visualized using three-dimensional molecular-graphics software.

### 4.8. Cell Culture and In Vitro Inflammatory Model

RAW264.7 murine macrophage cells were obtained from Procell (Wuhan, China, catalog number CL-0190). Cells were maintained and utilized for experiments within passages 3–8. Cells were seeded at a density of 2 × 10^5^ cells/mL and cultured overnight before experimental treatment. To establish an inflammatory cell model, RAW264.7 macrophages were stimulated with 100 ng/mL LPS (*E. coli* O111:B4, Sigma-Aldrich, St. Louis, MO, USA, catalog number L2630). Triptolide was administered concurrently with LPS stimulation, and the total treatment duration was 24 h. Dimethyl sulfoxide (DMSO) was used as vehicle control, and the final DMSO concentration was maintained below 0.1% (*v*/*v*).

### 4.9. Enzyme-Linked Immunosorbent Assay

After treatment, cell-culture supernatants were collected and centrifuged to remove cellular debris. Concentrations of TNF-α, IL-6, and IL-1β in culture supernatants were quantified using commercial ELISA kits from Multisciences (Lianke Biotech, Hangzhou, China). Catalog numbers: TNF-α (EK282/3-96), IL-6 (EK206/3-96), IL-1β (EK201B/3-96). The analytical detection range for each kit is as follows: TNF-α: 15.6–1000 pg/mL; IL-6: 7.8–500 pg/mL; IL-1β: 15.6–1000 pg/mL. Assays were performed strictly following the manufacturer’s instructions. Absorbance values were measured using a microplate reader. Cytokine concentrations were calculated against the standard curve supplied with each kit.

### 4.10. RNA Extraction and Quantitative Real-Time PCR

Total cellular RNA was extracted using TRIzol reagent (Invitrogen, Carlsbad, CA, USA, catalog number 15596026) according to the manufacturer’s protocol. RNA concentration and purity were assessed by spectrophotometry. Complementary DNA was synthesized from qualified total RNA using the PrimeScript RT reagent Kit with gDNA Eraser (Takara, Otsu, Shiga, Japan, catalog number RR047A). qRT-PCR amplification was carried out on the ABI 7500 Real-Time PCR System ABI 7500 Real-Time PCR System (Applied Biosystems, Foster City, CA, USA).

Actb was selected as the reference gene. Actb exhibited stable expression across all experimental groups under our LPS- and triptolide-treatment conditions in this study. Relative gene expression levels were calculated using the 2^−^ΔΔCt comparative threshold-cycle method.

The primer sequences used for qRT-PCR are listed below:

S100a8: forward 5′-AAGGAGAAGGCATTGAAGG-3′, reverse 5′-GTGGTGCCCTTTGATGTT-3′;

S100a9: forward 5′-ATGAGCAACCTGGAGAAG-3′, reverse 5′-GGTGGTCTGGTTTGTGG-3′;

Fcer1g: forward 5′-CCTGAAGTGAAGGTGATG-3′, reverse 5′-GATGGTGATGGTGAAGGT-3′;

Fcgr3: forward 5′-TCCTGCTGTTGGTGATGT-3′, reverse 5′-GCTGGTGAAGGTGATGAT-3′;

Lyz2: forward 5′-GCCAAGGTCTACAATCGT-3′, reverse 5′-TGATGGTGTCTCTGGTGT-3′;

Ms4a7: forward 5′-GCTGAAGAAGGTGGAGAT-3′, reverse 5′-CAGGTGATGGTGATGGT-3′;

Actb: forward 5′-CATCCGTAAAGACCTCTATGCCAAC-3′, reverse 5′-ATGGAGCCACCGATCCACA-3′.

### 4.11. Statistical Analysis

All in vitro cell experiments were performed with 3 biologically independent replicates, and each biological replicate included 3 technical replicates. GraphPad Prism 9 software (GraphPad Software, San Diego, CA, USA, accessed on 10 September 2026) was used for statistical analysis. The Shapiro–Wilk test was applied to assess the normality of data distribution. For datasets with a normal distribution, one-way analysis of variance (one-way ANOVA) followed by Tukey’s post hoc test was used for multiple group comparisons. For non-normally distributed datasets, the Kruskal–Wallis test with Dunn’s post hoc test was adopted. All post hoc tests included built-in multiple-comparison correction. Values of *p* < 0.05 were considered statistically significant.

## 5. Conclusions

In summary, this integrative and exploratory study identified a shared myeloid inflammatory gene signature underlying MASLD and RA by analyzing public transcriptomic datasets. S100A8/A9 was predicted to serve as a potential common molecule linking inflammatory responses in the two diseases. In vitro experiments in RAW264.7 macrophages indicated that triptolide could suppress LPS-induced inflammatory activation, downregulating S100a8/S100a9 expression and the shared inflammatory signature; whether these effects depend specifically on the S100A8/A9 complex remains to be established. Nevertheless, we emphasize that S100A8/A9 as a shared therapeutic target remains a hypothesis that requires further in vivo and clinical validation. Further investigations are warranted to dissect the precise regulatory mechanisms and evaluate the translational potential of triptolide in relation to the S100A8/A9 axis in MASLD and RA.

## Data Availability

The data presented in this study are publicly available in the Gene Expression Omnibus (GEO) repository at https://www.ncbi.nlm.nih.gov/geo/, reference number GSE66676, GSE55235, GSE192504 and GSE129516, accessed on 10 September 2026. All downstream analysis codes generated during this study are available from the corresponding author upon reasonable request.

## Abbreviations

The following abbreviations are used in this manuscript:

MASLD	Metabolic dysfunction-associated steatotic liver disease
NAFLD	Nonalcoholic fatty liver disease
MASH	Metabolic dysfunction-associated steatohepatitis
NASH	Nonalcoholic steatohepatitis
RA	Rheumatoid arthritis
DEG	Differentially expressed gene
GO	Gene Ontology
KEGG	Kyoto Encyclopedia of Genes and Genomes
PPI	Protein–protein interaction
ssGSEA	Single-sample gene set enrichment analysis
scRNA-seq	Single-cell RNA sequencing
UMAP	Uniform Manifold Approximation and Projection
CIA	Collagen-induced arthritis
GEO	Gene Expression Omnibus
GWAS	Genome-wide association study
LPS	Lipopolysaccharide
ELISA	Enzyme-linked immunosorbent assay
qRT-PCR	Quantitative real-time polymerase chain reaction
SEM	Standard error of the mean
FDR	False discovery rate
TNF-alpha	Tumor necrosis factor-alpha
IL-1beta	Interleukin-1 beta
IL-6	Interleukin-6
TLR	Toll-like receptor
NF-kappaB	Nuclear factor kappa B
MAPK	Mitogen-activated protein kinase
FCER1G	Fc epsilon receptor gamma subunit
FCGR2A	Fc gamma receptor IIa
FCGR3A	Fc gamma receptor IIIa
S100A8/A9	S100 calcium-binding protein A8/A9 heterodimer
